# Diagnostic value of high‐risk human papillomavirus viral load on cervical lesion assessment and ASCUS triage

**DOI:** 10.1002/cam4.3653

**Published:** 2021-03-07

**Authors:** Min Wang, Bo Hou, Xianzhen Wang, Lu Han, Yulin Shi, Yi Zhang, Lili Zhang, Lili Liu, Fen Jin, Yi Zhang

**Affiliations:** ^1^ Shengjing Hospital of China Medical University Shenyang China; ^2^ Anshan Cancer Hospital Anshan China; ^3^ The Second Hospital of Chaoyang Liaoning China; ^4^ Dalian Maternal and Child Care Hospital Dalian China; ^5^ Shenyang Women's and Children's Hospital Shenyang China; ^6^ Women's and Children's Hospital of Yingkou City Yingkou China; ^7^ The Second People's Hospital and Gynecology and Obstetrics Hospital of Fuxin City Fuxin China; ^8^ The First Affiliated Hospital of Jinzhou Medical University Jinzhou China; ^9^ Dalian Municipal Women's and Children's Medical Center Dalian China; ^10^ The First Affiliated Hospital of China Medical University Shenyang China

**Keywords:** ASCUS, cervical lesions, HR‐HPV viral load

## Abstract

This study aims to evaluate HR‐HPV viral load in the cervical lesion assessment and its diagnostic value on the triage of ASCUS. The three‐step protocol for cervical cancer screening was carried out in 5171 patients from June 2017 to August 2019, and 1620 histopathological results were obtained. The positive rate of HR‐HPV and TCT increased with the aggravation of pathological grades of cervical lesions. The sensitivity and specificity of HR‐HPV (DH3) to detect CIN II+ were 91.91% and 84.46%, respectively. In comparison, the corresponding results of the cytology test were 80.51% and 83.12%. HPV16/18 viral load was positively correlated with the grade of cervical lesions (*p* < 0.001, r = 0.321). The diagnostic efficiency of AUC by applying HPV16/18 viral load was 0.682 for the diagnosis of CIN II+. The optimal HPV16/18 viral load for predicting CIN II+ was 6.80 RLU/CO (relative light units/cut‐off), with corresponding sensitivity of 48.6%, specificity of 79.7%, and Youden index of 0.283. In the ASCUS population, viral loads were statistically different in HPV16/18 and the other 12 HR‐HPV when compared cervicitis group with CIN I group and CIN II+ group (all *p* < 0.05). Statistical differences were detected concerning HPV16/18 viral load, contact bleeding status, and smoking status when compared cervicitis group with CIN I group and CIN II+ group (*p* < 0.05), with a corresponding odds ratio of 1.004, 1.533, and 5.513, respectively. Our findings suggest that HR‐HPV viral load can be regarded as a useful tool to predict the grade of cervical lesions for ASCUS triage.

ClinicalTrials.gov ID: NCT03178136.

## Graphical abstract

This study which involves 5171 patients is focusing on high‐risk HPV viral load and cervical lesions. It is the first research on the diagnostic value of high‐risk HPV viral load by using DALTONbio hybrid capture 3. Our findings suggest that HPV16/18 viral load has good diagnostic efficacy and specificity in predicting CIN II + lesions. HR‐HPV viral load detection can be used to screen the high‐risk population with CIN II + lesion to the greatest extent.

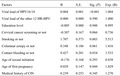

## 1 INTRODUCTION

Cervical cancer is one of the most common gynecologic malignant tumors that threaten the health of women. In China, cervical cancer has been the second most common female malignant tumor, which is characterized by younger onset and annual increase in its incidence.^1^

According to prior research, human papillomavirus (HPV) infection is intimately associated with the occurrence and development of cervical lesions. HPV infection is mostly a transient or intermittent phenomenon, 90% of which can be resolved spontaneously within two years, or develop into persistent infection.^2^ Persistent HPV infection may induce virus replication and integration, which eventually lead to cervical intraepithelial neoplasia (CIN). CIN is generally divided into CIN I, CIN II, and CIN III by grade. CIN I have a high probability of natural regression, and CIN II + lesions refer to CIN II and above (CIN II, CIN III, and cervical cancer). The International Federation for Cervical Pathology and Colposcopy (IFCPC) in 2012 proposed a new classification of cervical pathology, including low‐grade squamous intraepithelial lesions (LSIL) and high‐grade squamous intraepithelial lesions (HSIL), of which LSIL is CINI and HSIL covers CIN II and CIN III. Massive studies have confirmed that CIN II and CIN III have a great risk of developing cervical invasive cancer. Accordingly, early diagnosis and timely treatment of CIN II + lesions via cervical cancer screening are particularly important to reduce the incidence and mortality of cervical cancer.

The internationally recognized three‐step protocol for cervical cancer screening includes primary screening, colposcopy, and histopathology. At present, the primary screening scheme is widely applied in China by HPV detection and Thinprep cytologic test (TCT) alone or jointly. HPV genotyping, HPV‐RNA detection, and HPV‐DNA viral load detection are commonly used in Chinese hospitals. For those with abnormal primary screening, the scope and extent of the lesions can be further determined by colposcopy. Therefore, histopathological examination is still the gold standard for diagnosis and treatment.

HPV infection is the most common reproductive tract infection in women of childbearing age. The 14 internationally recognized high‐risk HPV (HR‐HPV) types are 16, 18, 31, 33, 35, 39, 45, 51, 52, 56, 58, 59, 66, and 68. HPV16 and HPV18 are known as the most serious types, with 39%‐78% of cervical cancer reported to be attributed to these two types.^3^ American Society for Colposcopy and Cervical Pathology (ASCCP) suggested that patients with negative cytology and HPV16/18 infection should be directly examined by colposcopy, while cytology triage and follow‐up should be recommended for other high‐risk types of HPV infection, especially those with persistent infection. ASCUS (Atypical squamous cells of undetermined significance) is a common epithelial abnormality diagnosed in cytology laboratories. It is defined as more obvious changes in cell abnormality than the reactive change, but not reaches the degree of squamous intraepithelial lesions. It can be benign lesions or a potential malignant change with active hyperplasia. According to the ASCCP recommendation, patients with ASCUS can be treated with cervical cytology retest or HR‐HPV shunt. Meanwhile, colposcopy should be performed if the cytology retest result is ≥ASC or HPV positive. However, frequent follow‐up may aggravate the psychological and economic burden of patients and cause additional anxiety and fear. Besides, unnecessary colposcopy referral may increase patients' pain and lead to excessive diagnosis and treatment. Because of the extreme scarcity of high‐level cytological technicians and pathologists, the low accuracy of diagnosis may result in potential misdiagnosis or even missed diagnosis.

In view of these problems, there are many researches committed to identifying the precise screening indicators of cervical cancer with clinical application value in recent decades. HR‐HPV viral load is so far one of the most controversial indicators. Unlike previous studies of HPV viral load, the present study applied DALTONbio hybrid capture 3 (DH3) to detect the viral load of HPV16/18 and the other 12 types of HR‐HPV viruses. This study is expected to investigate the correlation between the viral load of high‐risk HPV and cervical lesion grades as well as its diagnostic value for the shunt management of ASCUS.

## 2 MATERIALS AND METHODS

### 2.1 Clinical data

From June 2017 to August 2019, eligible subjects were selected from 10 hospitals, including Shengjing Hospital of China Medical University, Anshan Cancer Hospital, The Second Hospital of Chaoyang, Dalian Maternal and Child Care Hospital, Shenyang Women's and Children's Hospital, Women's and children's hospital of Yingkou City, The Second People's Hospital and Gynecology and Obstetrics Hospital of Fuxin City, The First Affiliated Hospital of Jinzhou Medical University, Dalian Municipal Women's and Children's Medical Center, The First Affiliated Hospital of China Medical University. The inclusion criteria were women aged 25–65 years old, with a medical history of sexual behavior, which voluntarily underwent cervical cancer screening. Those with a history of other systemic tumors and hysterectomy were excluded from this study. Samples of cervical exfoliated cells were collected for HR‐HPV viral load test and TCT. Basic information of enrolled patients was collected to establish medical records. The study was reviewed and approved by the hospital ethics committee, and all the subjects gave written informed consent.

### 2.2 Detection methods

Non‐menstrual samples were collected from subjects without intercourse and other vaginal operations three days prior sampling. Cervical brush and sample preservation solution were used for sampling in each test, and the test steps were strictly operated in accordance with the instructions for each reagent.

1. HR‐HPV viral load test: the 14 types of HR‐HPV (including 16, 18, 31, 33, 35, 39, 45, 51, 52, 56, 58, 59, 66, and 68) were detected quantitatively by DH3 (Hangzhou DALTONbio Co., Ltd.). The test result of the viral load value was calculated as relative light units/cut‐off (RLU/CO) ratio. The cut‐off value is 1.0 pg/mL. The viral load value ≥1 indicated a HPV positive result.
2. TCT: the TBS (The Bethesda System) grading system was used to evaluate the results by two pathologists. The abnormal results included atypical squamous cells undetermined significance (ASCUS), atypical squamous cells‐cannot exclude HSIL (ASC‐H), low squamous intraepithelial lesion (LSIL), high squamous intraepithelial lesion (HSIL), squamous of cervical carcinoma (SCC), etc. Subjects with ASCUS and above were determined as TCT positive results.

Patients with positive HR‐HPV viral load and TCT results were further treated according to the routine diagnosis and treatment procedures. Pathological examination was further performed in patients with abnormal results of colposcopy, if necessary. The final diagnosis was based on pathological results.

### 2.3 Statistical analysis

The database is established by EpiData 3.1 software. All of the patient's relevant information and test results are inputted into the final database after a duplicate check. By SPSS 22.0 software, analysis of data is achieved by various statistical methods, including spearman rank correlation analysis, χ^2^ test for comparison of rates, multivariate logistic regression analysis, receiver operator characteristic (ROC) curve, etc. *p* < 0.05 meant that the difference is statistically significant.

## 3 RESULTS

### 3.1 General data

A total of 5171 patients were enrolled in this study, including 1620 patients with histopathological findings, with an average age of 41 ± 10 years old. As for the pathological results, there were 472 cases of chronic cervicitis, 604 cases of CIN I, 299 cases of CIN II, 215 cases of CIN III, and 30 cases of cervical cancer. Table 1 shows the distribution of HR‐HPV viral load, HPV16/18 viral load, and TCT positive results in different grades of cervical lesions.

**TABLE 1:** Distribution of HR‐HPV and TCT results in cervical lesions of different grades

Histopathological results	HR‐HPV positive	HPV16/18 positive	TCT positive
Chronic cervicitis (n = 472)	294 (62.29%)	99 (20.97%)	314 (66.53%)
CIN I (n = 604)	425 (70.36%)	142 (23.51%)	467 (77.32%)
CIN II (n = 299)	276 (92.31%)	130 (43.48%)	235 (78.60%)
CIN III (n = 215)	195 (90.70%)	137 (63.72%)	178 (82.79%)
Cervical cancer (n = 30)	29 (96.67%)	24 (80.00%)	25 (83.33%)
Total cases (n = 1,620)	1,219 (75.25%)	532 (32.84%)	1,219 (75.25%)

### 3.2 Relationship of HR‐HPV viral load and TCT results with cervical lesions

The positive rates of TCT in chronic cervicitis, CIN I, CIN II, CIN III, and cervical cancer were 66.53% (314/472), 77.32% (467/604), 78.60% (235/299), 82.79% (178/215), and 83.33% (25/30), respectively, showing an increased trend. Besides, there was a grade correlation between the results of TCT and the grade of cervical lesions (*p* < 0.001, r = 0.275). The sensitivity and specificity of TCT to detect CIN II+ were 80.51% and 83.12%, respectively..

A total of 1,219 cases were positive for the detection of HR‐HPV viral load. The sensitivity and specificity of HR‐HPV (DH3) to detect CIN II+ were 91.91% and 84.46%, respectively. The distribution of HR‐HPV viral load in cervical lesions is shown in Table 2. There was a statistically significant difference in the mean viral load of HPV16/18 and the other 12 types of HR‐HPV in each group (*p* < 0.001).

**TABLE 2:** Distribution of the median viral load of HR‐HPV in cervical lesions

Histopathological results	Median viral load of HPV16/18 (pg/ml)	Median viral load of the other 12 HR‐HPV (pg/ml)
Chronic cervicitis (n = 472)	0.22	14.045
CIN I (n = 604)	0.28	45.58
CIN II (n = 299)	0.78	35.29
CIN III (n = 215)	31.1	4.82
Cervical cancer (n = 30)	77.47	3.16
Significance (P)	<0.001	<0.001

Spearman rank correlation analysis showed that HPV16/18 viral load was correlated positively with the grade of the cervical lesion (*p* < 0.001, r = 0.321). In HPV16/18 positive cases, the HPV16/18 viral load was positively correlated with cervical lesion grades, regardless of the other 12 types of HR‐HPV results (*p* < 0.001, r = 0.308). In HPV16/18 negative and the other 12 types of HR‐HPV positive cases, the other 12 types of HR‐HPV viral load have a weak positive correlation with cervical lesion grades (*p* = 0.005, r = 0.106).

### 3.3 Monitoring effect of HR‐HPV viral loads in cervical lesions and its triaging effect on cytological abnormalities

ROC curve was generated from 1620 biopsy results. Chronic cervicitis and CIN I lesions were considered as negative events and CIN II+ as positive events. The diagnostic efficiency of AUC by applying HPV16/18 viral load was 0.682 for the diagnosis of high‐grade cervical lesions. The Youden index was further calculated according to the sensitivity and specificity of each possible cut‐off point in the statistical results. Using the largest tangent point of the Youden index as the critical point, the optimal viral load value for predicting CIN II + lesions was 6.80 RLU/CO, with corresponding sensitivity of 48.6%, specificity of 79.7%, and Youden index of 0.283.

Among 1620 subjects with biopsy results, there were 495 cases of ASCUS, 132 cases of ASC‐H, 397 cases of LSIL, 177 cases of HSIL, and 18 cases of AGC according to the results of TCT. There were significant differences in the distribution of HPV16/18 viral load and other 12 types of HR‐HPV viral loads in cervical lesions of different grades based on cervical cytology (all *p* < 0.001). Spearman rank correlation test showed that HPV16/18 viral load was weakly correlated with the abnormal grade of cervical cytology (*p* = 0.023, r = 0.066). Meanwhile, the other 12 types of HR‐HPV viral loads were positively correlated with abnormal grade of cervical cytology (*p* < 0.001, r = 0.113).

Among 495 ASCUS cases, there were 172 cases of chronic cervicitis, 206 cases of CIN I, 76 cases of CIN II, 37 cases of CIN III, and 4 cases of cervical cancer. Table 3 displays the distribution of HR‐HPV and HPV16/18 positive results in cervical lesions of different grades. The relationship between the HR‐HPV and CIN II + lesions was further analyzed in Tables 4, 5. Chronic cervicitis and CIN I lesions were considered as negative events and CIN II+ as positive events. The results indicated that there were significant differences in the viral load of HPV16/18 and the other 12 types of HR‐HPV when compared cervicitis group with CIN I lesion group (*p* < 0.001) and CIN II + lesion group (*p* < 0.001). Besides, the sensitivity of HR‐HPV viral load was 94.87% to diagnose CIN II+lesions.

**TABLE 3:** Distribution of HR‐HPV and HPV16/18 genotyping in cervical lesions of different grades among ASCUS population

Histopathological results	HR‐HPV positive	HPV16/18 positive
Chronic cervicitis (n = 172)	88 (51.16%)	28 (16.28%)
CIN I (n = 206)	139 (67.48%)	40 (19.42%)
CIN II (n = 76)	74 (97.37%)	32 (42.11%)
CIN III (n = 37)	33 (89.19%)	24 (64.86%)
Cervical cancer (n = 4)	4 (100.00%)	4 (100.00%)
Total (n = 495)	338 (68.28%)	128 (25.86%)

**TABLE 4:** Comparison of HPV16/18 viral load and cervical pathology among ASCUS population

Viral load of HPV16/18	Histopathological results		Total
	CIN2+	CIN1‐	
Positive	60 (46.88%)	68 (53.12%)	128
Negative	57 (15.53%)	310 (84.47%)	367
Total	117 (23.64%)	378 (76.36%)	495

**TABLE 5:** Comparison of the other 12 HR‐HPV and cervical pathology among ASCUS population

Viral load of the other 12 HR‐HPV	Histopathological results		Total
	CIN2+	CIN1‐	
Positive	89 (30.27%)	205 (69.73%)	294
Negative	28 (13.93%)	173 (86.07%)	201
Total	117 (23.64%)	378 (76.36%)	495

### 3.4 Logistic regression analysis of the risk factors of CIN II + LESIONS

On the basis of prior literature guideline and professional knowledge, the factors that may be strongly correlated with the occurrence and development of cervical lesions were screened for logistic regression analysis. Relevant factors included 10 variables such as educational level, cervical cancer screening, smoking, columnar ectopy, contact bleeding, age of sexual initiation, age of first pregnancy, medical history of CIN, and two HR‐HPV viral loads. Logistic regression analysis was carried out for the variables with *p* < 0.2 in univariate analysis (Table 6). According to our results, there were statistical differences in HPV16/18 viral load, contact bleeding, and smoking when compared cervicitis group and CIN I lesion group with CIN II + lesion group (*p* < 0.05), with corresponding OR were 1.004, 1.533, and 5.513, respectively. However, no statistical difference was found in the viral load of the other 12 HR‐HPV, education level, cervical cancer screening, columnar ectopy, age of sexual initiation, age of first pregnancy, and medical history of CIN (*p* > 0.05).

**TABLE 6:** Analysis of risk factors related to CIN II + lesions

Factors	B	S.E.	Sig. (*P*)	Exp. (B)
Viral load of HPV16/18	0.004	0.001	<0.001	1.004
Viral load of the other 12 HR‐HPV	0.000	0.000	0.590	1.000
Education level	−0.005	0.080	0.948	0.995
Cervical cancer screening or not	−0.307	0.167	0.066	0.736
Smoking or not	1.707	0.573	0.003	5.513
Columnar ectopy or not	0.348	0.186	0.061	1.416
Contact bleeding or not	0.427	0.201	0.034	1.533
Age of sexual initiation	−0.176	0.168	0.293	0.838
Age of first pregnancy	0.029	0.147	0.844	1.029
Medical history of CIN	0.239	0.253	0.345	1.270

## 4 DISCUSSION

Massive studies have confirmed that HR‐HPV infection is a necessary condition for the presence of cervical precancerous lesions and cervical cancer.^4^, ^5^ Meanwhile, supported by a large number of clinical studies, it will take about 7–12 years for HR‐HPV infection to develop into cervical carcinoma in situ. Regarding this, early diagnosis and timely treatment of CIN II + lesions become particularly important for cervical cancer screening.

Currently, TCT combined with HPV test is used in Chinese cervical cancer screening program. Abundant evidence^6^ revealed that there was an increasing trend in the HPV infection rate and TCT positive rate along with the increase of cervical lesion grades. The relative risk of HPV infection and cervical cancer reached 307.5. The results of our study are consistent with previous studies. The sensitivity and specificity of HR‐HPV (DH3) to detect CIN II+ were 91.91% and 84.46%, respectively. In comparison, the corresponding results of the cytology test were 80.51% and 83.12%. Meanwhile, there was a grade correlation between TCT results and cervical lesion grades, with the correlation coefficient of r = 0.275. According to these results, HR‐HPV load had a similar specificity but higher sensitivity than TCT in diagnosing CIN II+ lesions, suggesting that the initial screening of cervical cancer by HR‐HPV viral load can reduce the missed diagnosis of high‐grade cervical lesions and to improve the accuracy of screening.

In virus infection‐related tumors, high viral load has been reported to be significantly related to the risk of carcinogenesis. For example, Epstein‐Barr virus DNA load was intimately correlated with the occurrence and development of nasopharyngeal carcinoma.^7^ However, it remains controversial with respect to the relationship between HPV viral load and cervical lesions. Different HPV types may concern the correlation between HR‐HPV viral load and the severity of cervical lesions.^8^, ^9^ There is a gradual deepening in cervical lesion grades with the increase of HPV16 and HPV18 viral loads, accompanied by a strong correlation between them. Depuydt^10^ et al. supported that pathological changes in CIN III might be caused by the stable increase of specific HPV load, and the detection of specific HPV load might have a prediction function for the early CIN III. Park JY^11^ and Xi LF et al,^12^ verified in their study that HPV18 viral load was lower in cervical precancerous lesions, and increased significantly in cervical cancer. In our study, a similar finding was revealed that HPV16/18 viral load increased with the grade of cervical lesions, with the correlation coefficient of 0.321. It suggested that HPV16/18 viral load may be applied as a diagnostic index to predict the grade of cervical lesions. Furthermore, the ROC curve was generated to evaluate the diagnostic efficacy and threshold of HPV16/18 viral load on cervical lesions. It was found that the area under the curve (AUC) was 0.682, and the optimal viral load cut‐off value of predicting CIN II + cervical lesions was 6.80 RLU/CO, with corresponding sensitivity of 48.6% and specificity of 79.7%. These results indicated that HPV16/18 viral load may have good diagnostic efficacy and specificity in predicting CIN II + lesions, which may contribute to reducing unnecessary colposcopy referral and decreasing the fear and anxiety of patients with transient HPV infection. Dong B et al.^13^ reported in their research that the viral load of HPV18 in CIN II + lesions was higher than that in low‐grade cervical lesions. Besides, the viral load of HPV31, 33, 52, and 58 was positively correlated with the grade of cervical lesions, while HPV45, 56, and 59 showed no obvious correlation with the grade of cervical lesions. Another research^14^ proposed that the high‐risk HPV viral load except HPV16 and 18 may indicate the potential high risk of CIN II + lesions. In our study, there was no grade correlation between the other 12 types of HR‐HPV viral loads and cervical lesions. This may be due to cervical lesions caused by the other 12 types of HR‐HPV infection are primarily correlated with the transcription level of HPV and integration status of HPV, and present a slight correlation with the copy number of HPV.^15^ However, its positive status and viral load value are still of great significance for the diagnosis of CIN II + lesions. With the increase of the other 12 types of HPV viral loads, the incidence of CIN II + lesion showed an upward trend despite no significant increase in proportion. It suggests that the risk of high viral load value still deserves the attention of clinicians. Furthermore, certain reports^14^ argue that there was insufficient evidence to determine the correlation between HR‐HPV viral load and the severity of disease. In their report, HR‐HPV viral load had no obvious numerical limit from inflammation to CIN III according to the results of the pathological examination, indicating that HR‐HPV viral load cannot be used as an indicator for predicting cervical lesions. These different results may be related to the different number of exfoliated cells in samples and the size of cervical lesions,^16^ as well as the inconsistencies in the criteria of dividing the viral load of HR‐HPV.

ASCUS is the most common abnormal diagnosis result in TCT, and its proportion is much higher than other results. ASCUS is defined as more obvious changes in cell abnormality than the reactive change, but not reaches the degree of squamous intraepithelial lesions. It can be a benign change or a potential malignant change with active hyperplasia. In a study carried out by Yarandi F et al.,^17^ the diagnosis rate of ASCUS was 3–10% in liquid‐based TCT of cervix, which could range from normal cervix to histopathological cervical cancer. Besides, the discovery of a considerable proportion of ASCUS may inhibit the diagnosis of high‐grade lesions such as CIN II, CIN III, and even invasive cervical cancer. Furthermore, Li X^18^ showed that the detection rate of CIN II was 13.55% in 251 cases with ASCUS, while the detection rate of high‐grade lesion was 7.3% in 463 cases of ASCUS as reported by Li SR.^19^ ASCUS is an exclusive diagnosis rather than a clear diagnosis of lesions. It may be benign or potentially malignant, with great differences in clinical treatment. In our study, there was a significant difference concerning the distribution of HPV16/18 viral load and the other 12 types of HR‐HPV viral loads in cervical lesions of different grades based on cervical cytology. A positive correlation was found between cervical lesions of different grades and HPV16/18 viral load. Meanwhile, there was a significant difference in HPV16/18 viral load and the other 12 types of HR‐HPV viral loads when compared cervicitis group with CIN I lesion group and CIN II+ group, suggesting that HR‐HPV viral load value can exert an indicator for triage of ASCUS. Previous studies have confirmed that HR‐HPV DNA testing is a feasible method for the triage of ASCUS. In our study, we used viral load rather than amplification cycles to analyze the role of HPV DNA detection in ASCUS shunt. The DH3 is a nucleic acid hybridization assay with signal amplification using microplate chemiluminescent detection. It is able to detect the 2 types (16 and 18) and 12 types (31, 33, 35, 39, 45, 51, 52, 56, 58, 59, 66, and 68) of HR‐HPV DNA in two microplates separately. Using this hybrid capture technology based HPV16/18 genotyping test, we concluded that HPV16/18 viral load rather than the other 12 types of HR‐HPV viral loads detection can be used to triage the CIN II + lesion in the ASCUS population. The patients who exceed the clinical HPV16/18 viral load threshold could be referred to colposcopy directly. Other HPV positive patients (including HPV16/18 positive but viral load below the threshold) could be followed up for repeat testing. HR‐HPV viral load is expected to improve the cervical cancer screening efficiency for clinical application; however, further research is still needed to verify the application of HR‐HPV viral load.

According to the logistic regression analysis results, there were statistical differences in HPV16/18 viral load, contact bleeding, and smoking when compared cervicitis group and CIN I lesion group with CIN II + lesion group (*p* < 0.05), with corresponding OR of 1.004, 1.533 and 5.513, respectively. However, the viral load of the other 12 types of HR‐HPV, education level, cervical cancer screening history, columnar ectopy, age of sexual initiation, age of first pregnancy, medical history of CIN were not risk factors of high‐grade cervical lesions. It is known that multiple factors seem to intervene in cervical lesions.^20^ However, contact bleeding is more likely a symptom of cervical lesions than a cause. Smoking is closely related to other confounding factors, like unfavorable psychosocial events, local immunosuppression, and nutrition, which got a difficult epidemiologic evaluation of smoking role on cervical carcinogenesis. Our results indicated that smoking habits should be taken into account in clinical practice and in research concerning. In addition, this research included woman aged 25–65 years old, further study should be investigated in younger age group with an expanded sample size.

In summary, HR‐HPV (especially HPV16/18) viral load value may be considered as a potential indicator to predict cervical lesion grade and ASCUS triage management. HR‐HPV viral load is expected to be applied in clinical cervical cancer screening, which will make the screening and ASCUS triage more effective and precise.

## 5 Conflict of interest statement

There is no conflict of interest in this study.

## Data Availability

All data included in this study are available on reasonable request from the corresponding author.
